# Taxonomic evaluation of selected *Ganoderma* species and database sequence validation

**DOI:** 10.7717/peerj.3596

**Published:** 2017-07-27

**Authors:** Suldbold Jargalmaa, John A. Eimes, Myung Soo Park, Jae Young Park, Seung-Yoon Oh, Young Woon Lim

**Affiliations:** 1 School of Biological Sciences, Seoul National University, Seoul, South Korea; 2 University College, Sungkyunkwan University, Suwon, South Korea

**Keywords:** *Ganoderma*, Polypores, Medicinal fungi, Genbank sequence validation

## Abstract

Species in the genus *Ganoderma* include several ecologically important and pathogenic fungal species whose medicinal and economic value is substantial. Due to the highly similar morphological features within the *Ganoderma*, identification of species has relied heavily on DNA sequencing using BLAST searches, which are only reliable if the GenBank submissions are accurately labeled. In this study, we examined 113 specimens collected from 1969 to 2016 from various regions in Korea using morphological features and multigene analysis (internal transcribed spacer, translation elongation factor 1-*α*, and the second largest subunit of RNA polymerase II). These specimens were identified as four *Ganoderma* species: *G. sichuanense*, *G.* cf. *adspersum*, *G.* cf. *applanatum*, and *G.* cf. *gibbosum*. With the exception of *G. sichuanense*, these species were difficult to distinguish based solely on morphological features. However, phylogenetic analysis at three different loci yielded concordant phylogenetic information, and supported the four species distinctions with high bootstrap support. A survey of over 600 *Ganoderma* sequences available on GenBank revealed that 65% of sequences were either misidentified or ambiguously labeled. Here, we suggest corrected annotations for GenBank sequences based on our phylogenetic validation and provide updated global distribution patterns for these *Ganoderma* species.

## Introduction

Fungal species in the genus *Ganoderma* Karst. (Ganodermataceae, Polyporales) include ecologically important wood decay fungi of which some species are a well-known component of traditional Asian medicine. Several species of *Ganoderma* have been reported to cause diseases associated with trees, including basal stem rot disease in oil palm caused by *G. boninense* (Susanto, Sudharto & Purba, 2005), and root-rot disease of *Acacia* trees caused by *G. steyaertanum, G. mastoporum,* and *G. philippii* (Glen et al., 2009). Despite the pathogenic nature of many *Ganoderma* species, many species, especially taxa identified as *G. lucidum* in Asia, are believed to possess medicinal characteristics, and have been used in traditional Asian medicine for millennia (Bishop et al., 2015). *Ganoderma* byproducts are increasingly being used in western medicine and related health industries, and the most recent estimate of the annual economic value of *Ganoderma* byproducts (calculated in 1995) was ∼1.6 billion USD (Chang & Buswell, 1999); adjusting for inflation and economic growth, this annual economic value is likely several billion dollars in 2017. As our understanding of the biochemistry and genetics of *Ganoderma* biocompounds increases in tandem with increasing medicinal and economic demand for these byproducts, accurate identification of *Ganoderma* species is critical.

*Ganoderma* is likely a relatively young genus, originating in the tropics and recently expanding its range into temperate zones. The estimated number of *Ganoderma* species ranges from 250 to >400 (Moncalvo, Wang & Hseu, 1995; Richter et al., 2015). The morphology of *Ganoderma* species is characterized by a crusty or shiny pileus surface and a two-layered basidiospore wall with a truncated apex. Due to the high similarity of basidiocarp features, it is likely that the *Ganoderma* is the most difficult genus to accurately identify to species of all polypores (Ryvarden & Gilbertson, 1993). In fact, the *Ganoderma* have been described as being in a state of “taxonomic chaos” (Ryvarden, 1991). Indeed, the wide range of estimates for the number of *Ganoderma* species exemplifies the ambiguity that permeates the taxonomy of this genus. Early efforts to apply molecular markers toward the resolution of *Ganoderma* taxonomy used sequences from internal transcribed spacer (ITS) and partial large subunit rDNA (Moncalvo et al., 1994; Moncalvo, Wang & Hseu, 1995; Moncalvo, 2000) and nearly complete small subunit rDNA sequences (Hong & Jung, 2004; Douanla-Meli & Langer, 2009). More recently, Wang et al. (2012) assessed the identification of European *G. lucidum* (a species originally described from England) and East Asia *G. lucidum* using three loci and determined that the East Asia samples were genetically distinct from their European counterparts and conspecific with *G. sichuanense.* Further complicating matters, Cao, Wu & Dai (2012) combined morphological characters with phylogenetic analyses of six loci and proposed naming East Asian *G. lucidum* as *G. lingzhi*; however, *G. lingzhi* is regarded as a synonym of *G. sichuanense*, the name proposed in 1983 (Wang et al., 2012). Following the rules of fungal nomenclature, the name *G. sichuanense* should be given preference over any synonyms (Richter et al., 2015).

The dramatic increase in available DNA sequences from molecular phylogenetic studies has helped resolve the taxonomy of numerous fungal groups. Sequences from the ITS have been particularly useful in the recent development of DNA barcoding of the fungi (Schoch et al., 2012), although other loci such as translation elongation factor 1-α (*tef1-α*) and the second largest subunit of RNA polymerase II (*rpb2*) (Liu, Whelen & Hall, 1999; Matheny et al., 2007) have been instrumental in resolving ambiguous evolutionary relationships among *Ganoderma* species. While molecular advances have in general led to improvements in phylogenetics and taxonomy, the relative ease and speed of DNA barcoding also has the potential to increase the confusion often associated with fungal taxonomy. Up to 20% of designated sequences in public databases may be erroneous because of improper species identification (Bridge et al., 2003; Vilgalys, 2003; Nilsson et al., 2006). This problem is acute within the genus *Ganoderma* due to the absence of reliable morphological characteristics, a high rate of synonymous classification, and incorrect taxonomic assignments in public databases. The combination of morphology and molecular analysis (ideally multi-locus) can resolve the issues associated with this growing problem of misidentified sequence in public databases (Jung et al., 2014).

Sequencing costs have recently fallen precipitously as has the speed and ease with which specimens can be identified via sequencing. As a result, an increasing number of non-specialists, including edible mushroom cultivators as well as collectors and re-sellers of medicinal fungi, have relied on commercial sequencing companies to identify specimens. These identifications are often made using BLAST searches, which are only reliable if the GenBank submissions are accurately labeled. Thus, a re-evaluation of *Ganoderma* species that are endemic to Korea is needed and the accuracy of sequence databases such as GenBank should be investigated.

In Korea, the first report of *Ganoderma lucidum* (reported as *Fomes japonicus*) was 1934 (Murata, 1934). The classification system of *Ganoderma* proposed by Imazeki (1952) divided the *Ganoderma* into two subgenera, with laccate species (including *G. lucidum*) in the subgenus *Ganoderma* and non-laccate species (including *E. applanatum*) in the subgenus *Elfvingia*. To date, five *Ganoderma* species, *G. applanatum, G. lipsiense*, *G. lucidum G. neojaponicum*, and *G. tsugae*, have been recorded in Korea (Kaburagi, 1940; Lee, 1981; Kang, 1991; Kwon et al., 2016). *G. lipsiense*, however, is synonymous with *G. applanatum* (Moncalvo & Ryvarden, 1997), therefore only four *Ganoderma* species were known in Korea. Importantly, most Korean *Ganoderma* species were reported solely on basidiocarp morphology without detailed descriptions or molecular data.

In this study, our main objective was to build a Korean *Ganoderma* inventory using morphology and molecular analysis and to correct species misidentifications in GenBank. In addition, we provide updated global distribution patterns of these four *Ganoderma* species using newly validated GenBank sequences.

**Table 1:** Representative *Ganoderma* specimens from the Seoul National University Fungus Collection (SFC) used in this study.

Species	Specimen no.	Collection sites		Accession number		
		Locality	Latitude/Longitude	ITS	*rpb2*	*tef1-α*
*G. sichuanense*	SFC20120721-08	Gimpo-si, Gyeonggi-do	37°36′08.43″N/126°46′33.51″E	KY364244		
	SFC20150624-06	Pohang-si, Gyeongsangbuk-do	36°04′20.02″N/129°12′36.56″E	KY364245	KY393267	KY393279
	SFC20150630-14	Jongno-gu, Seoul	37°34′28.50″N/126°59′38.92″E	KY364246	KY393268	KY393280
	SFC20150812-48	Jongno-gu, Seoul	37°34′28.30″N/126°59′44.28″E	KY364247		KY393281
	SFC20150918-07	Jongno-gu, Seoul	37°34′26.70″N/126°59′36.12″E	KY364248	KY393269	KY393282
	SFC20160315-03	Yangyang-gun, Gangwon-do	38°07′17.46″N/128°33′06.78″E	KY364249		KY393283
	SFC20160420-01	–^a^		KY364250		
*G.* cf. *adspersum*	SFC20141001-16	Inje-gun, Gangwon-do	37°57′11.80″N/128°19′24.52″E	KY364251	KY393270	KY393284
	SFC20141001-22	Inje-gun, Gangwon-do	37°57′02.55″N/128°19′29.46″E	KY364252	KY393271	KY393285
	SFC20140701-31	Inje-gun, Gangwon-do	37°56′50.06″N/128°19′47.85″E	KY364253		
	SFC20160115-20	Yangpyeong-gun, Gyeonggi-do	37°29′20.09″N/127°36′34.14″E	KY364254	KY393272	KY393286
*G.* cf. *applanatum*	SFC20141001-24	Inje-gun, Gangwon-do	37°56′46.46″N/128°20′00.98″E	KY364255	KY393273	KY393287
	SFC20141001-25	Inje-gun, Gangwon-do	37°57′13.39″N/128°19′16.80″E	KY364256		
	SFC20141012-02	Inje-gun, Gangwon-do	37°57′12.84″N/128°19′18.18″E	KY364257		
	SFC20150930-02	Inje-gun, Gangwon-do	38°07′30.12″N/128°12′10.08″E	KY364258	KY393274	KY393288
*G.* cf. *gibbosum*	SFC20130404-21	Sangju-si, Gyeongsangbuk-do	36°31′47.93″N/128°04′27.06″E	KY364259		
	SFC20140702-12	Seogwipo-si, Jeju-do	33°14′54.58″N/126°21′03.42″E	KY364260	KY393275	
	SFC20140703-17	Jeju-si, Jeju-do	33°26′24.63″N/126°37′39.49″E	KY364261		
	SFC20150418-05	Gwanak-gu, Seoul	37°27′21.15″N/126°56′57.18″E	KY364262		
	SFC20150612-11	Donghae-si, Gangwon-do	37°27′51.09″N/129°01′00.16″E	KY364263		
	SFC20150630-23	Jongno-gu, Seoul	37°34′24.07″N/126°59′36.17″E	KY364264	KY393276	KY393289
	SFC20150701-06	Jeju-si, Jeju-do	33°19′31.62″N/126°16′50.62″E	KY364265		
	SFC20150723-01	Jongno-gu, Seoul	37°34′22.66″N/126°59′37.49″E	KY364266		
	SFC20150812-02	Jongno-gu, Seoul	37°34′21.62″N/126°59′38.18″E	KY364267		
	SFC20150812-35	Jongno-gu, Seoul	37°34′20.40″N/126°59′45.21″E	KY364268		
	SFC20150812-36	Jongno-gu, Seoul	37°34′27.81″N/126°59′44.59″E	KY364269		
	SFC20150918-03	Jongno-gu, Seoul	37°34′24.63″N/126°59′39.11″E	KY364270	KY393277	KY393290
	SFC20150918-08	Jongno-gu, Seoul	37°34′20.23″N/126°59′44.05″E	KY364271	KY393278	KY393291
	SFC20160713-09	Jeju-si, Jeju-do	33°29′26.67″N/126°36′08.87″E	KY364272		

**Notes.**

a No information.

## Materials and Methods

### Sample collection and morphological analysis

Specimens were collected from 1969 to 2016 from various regions in Korea and stored at the Seoul National University Fungus Collection (SFC) and Korea Mushroom Resource Bank (KMRB). These specimens were initially identified as *G. lucidum, G. neojaponicum*, and *G. applanatum* (Table S1). Because the complex characteristics of the basidiocarps of *Ganoderma* have contributed to confusion in the taxonomy of this genus, we sorted specimens using macro- and micro-morphological observations (Gilbertson & Ryvarden, 1986; Cao, Wu & Dai, 2012). Initially, three morphological features were observed for 113 specimens: shape of basidiocarp, pore number per mm at hymenophore, and basidiospore size. Pore number was calculated as the mean of five 1 mm transects across hymenium. In order to observe basidiospores, slide preparations mounted in 3% KOH were made from dried tissue for each specimen and examined with a Nikon 80i light microscope (Nikon, Tokyo, Japan).

### DNA extraction, amplification, and sequencing

A total of 29 recently collected Korean specimens were chosen for DNA sequencing (Table 1). Genomic DNA was extracted using a modified CTAB extraction protocol (Rogers & Bendich, 1994). The ITS region, partial *tef1-α,* and partial *rpb2* regions were amplified using the primers ITS1F/ITS4b (Gardes & Bruns, 1993), EF1-983F/EF1-2218R (Rehner & Buckley, 2005), and fRPB2-5F/bRPB2-7R2 (Liu, Whelen & Hall, 1999; Matheny et al., 2007), respectively. PCRs were performed on a C1000TM thermal cycler (Bio-Rad, Richmond, CA, USA) using AccuPower PCR premix (Bioneer Co., Daejeon, Korea) according to the methods described in Park et al. (2013). PCR products were electrophoresed through a 1% agarose gel stained with EcoDye DNA staining solution (SolGent Co., Daejeon, Korea) and purified using the Expin™ PCR Purification Kit (GeneAll Biotechnology, Seoul, Korea) according to the manufacturer’s instructions. DNA sequencing was performed at Macrogen (Seoul, Korea) using an ABI3700 automated DNA sequencer. Sequences obtained from specimens were proofread using chromatograms in MEGA v. 6 (Tamura et al., 2013).

### Phylogenetic analysis

Phylogenetic analysis was carried out in two steps. First, phylogenetic trees using ITS, *tef1-α*, and *rpb2* sequences were constructed using only specimens of Korean *Ganoderma* species. Second, we downloaded all *Ganoderma* sequences obtained from the search query “*Ganoderma*” in GenBank. SFC amplicon sequences of ITS, *tef1-α*, and *rpb2* were aligned with *Ganoderma* sequences downloaded from GenBank using the default settings of MAFFT v.7 (Katoh & Standley, 2013). Maximum likelihood (ML) trees were constructed with RAxML 8.0.2 (Stamatakis, 2014) using the GTRGAMMA model of evolution and 1,000 bootstrap replicates. *Coriolopsis* cf. *caperata* was used as an outgroup for all three phylogenetic trees (Binder et al., 2013).

### Validation and distribution of GenBank *Ganoderma* sequences

We used BLAST to validate *Ganoderma* sequences in GenBank. GenBank sequences for *G. sichuanense* were available for all three loci (ITS, *tef1-α*, and *rpb2*); however, there were insufficient sequences for the other species at the *tef1-α* and *rpb2* loci. Thus, our validation for these species is limited to the ITS locus. ITS sequences from each Korean species were used for the BLAST searches and sequences were downloaded based on similarity and coverage. We downloaded all sequences that had a similarity of >90% at the ITS. We excluded short sequences by removing those that had coverage of <50%. Neighbor Joining (NJ) analyses were performed using these sequences to determine the correct sequence identity for each *Ganoderma* species. NJ trees were constructed with MEGA v. 6, using the Kimura 2-parameter model and 1,000 bootstrap replicates. All work with GenBank was performed on September 20, 2016.

We used the validated sequence information of four *Ganoderma* species to generate a map of the global distribution. Distribution information of each species was extracted from published papers and direct GenBank submissions.

**Figure 1:**
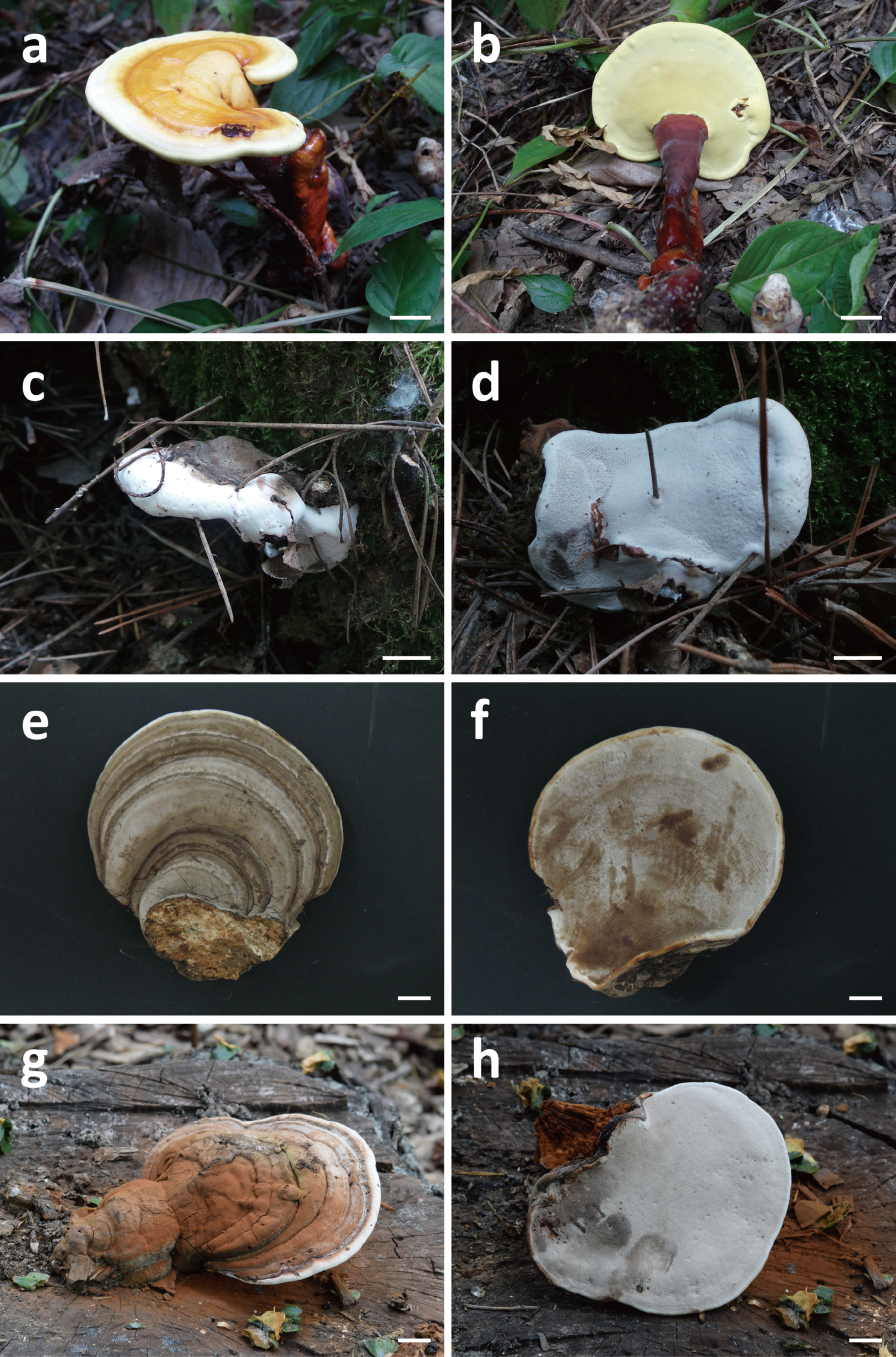
Basidiocarps of *Ganoderma*; *G. sichuanense* (A–B), *G.* cf. *adspersum* (C–D), *G.* cf. *applanatum* (E–F), and *G.* cf. *gibbosum* (G–H). *Scale bars*: (A–H) = 1 cm.

**Figure 2:**
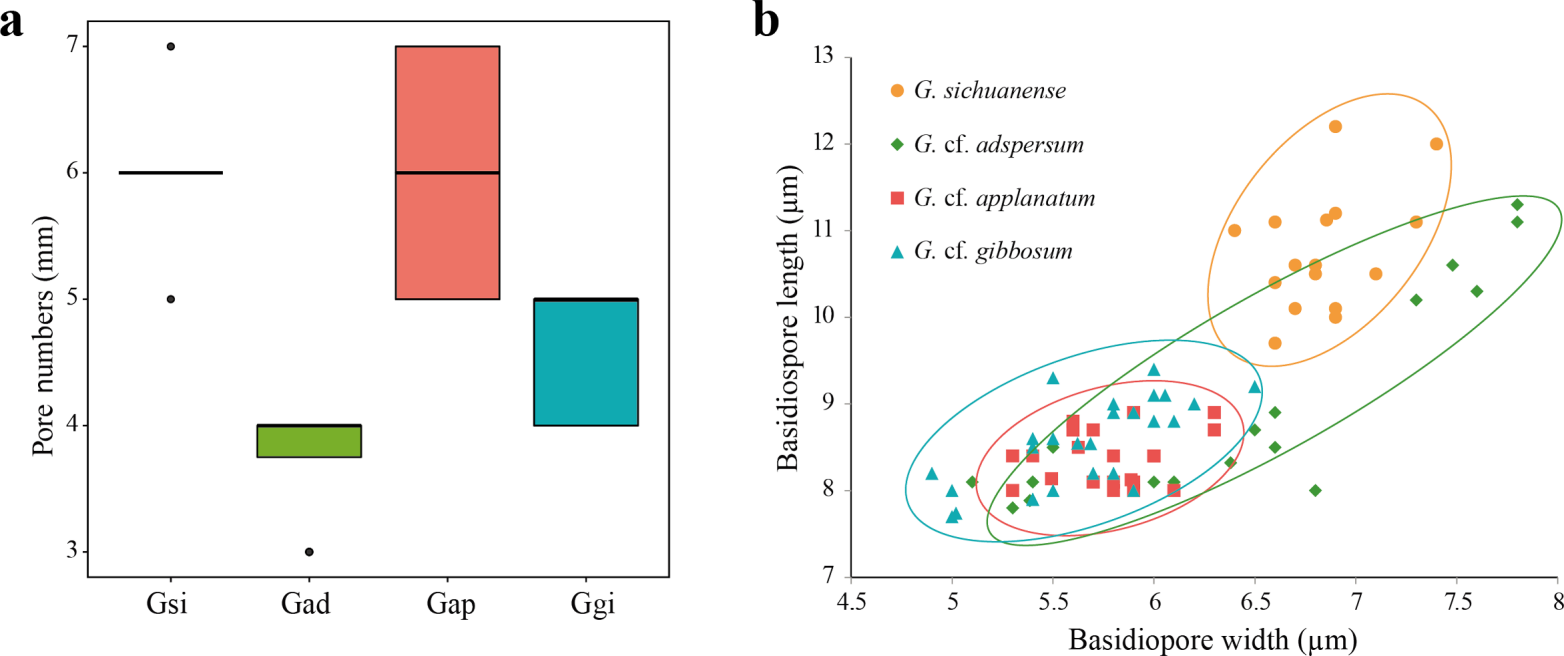
(A) Box plot representing pore number per mm of four *Ganoderma* species: *G. sichuanense* (Gsi), *G.* cf. *adspersum* (Gad), *G.* cf. *applanatum* (Gap), and *G.* cf. *gibbosum* (Ggi). Boxes represent the interquartile range between first quartile and third quartile. Bold line in the box is the median and filled circles represent individual outlying data points. (B) Scatter plot of basidiospore size among the four species (mm). Four samples were observed for each species.

## Results

### Evaluation of *Ganoderma* specimens based on morphological and molecular analyses

All 113 specimens identified as *Ganoderma* were used in the preliminary portion of this study. These samples were reexamined based on distinguishable morphological characters. First, specimens with laccate basidiocarps and long stipes were distinguished from other specimens with non-laccate basidiocarps. Laccate specimens initially identified as *G. lucidum*, *G. lingzhi* and *G. neojaponicum* were identified as *G. sichuanense* using molecular analysis based on ITS, *tef-1*, and *rpb2*. Basidiocarps were reniform to circular with long subcylindrical stipe (Figs. 1A and 1B), had circular or angular pores that were at a density of 5–6 per mm (Fig. 2A), and basidiospore size was (9.7) 10.4–11.1 (12.2) × (6.4) 6.6–6.9 (7.4) μm (Fig. 2B).

While all non-laccate specimens were similar to *G. applanatum,* they could be separated into three different morphology types. Type A specimens had sessile basidiocarps, and were attached directly to the tree at its base with no stipe (Figs. 1C and 1D). Pore number was 3–4 per mm (Fig. 2A) and basidiospore size range was (7.8) 8.3–10.6 (11.3) × (5.1) 5.4–7.4 (7.8) μm (Fig. 2B). Type B specimens had sessile basidiocarps with no stipes (Figs. 1E and 1F), a pore number range of 5–7 per mm (Fig. 2A), and basidiospore size of (8.0) 8.1–8.5 (8.9) × (5.3) 5.4–5.8 (6.3) μm (Fig. 2B). The basidiocarps of type C specimens were attached to broad-leaved tree stumps with short stipes (Figs. 1G and 1H), a pore number range of 4–5 per mm (Fig. 2A), and basidiospore size of (7.7) 8.5–9.2 (9.4) × (4.9) 5.6–6.0 (6.5) μm (Fig. 2B).

**Figure 3:**
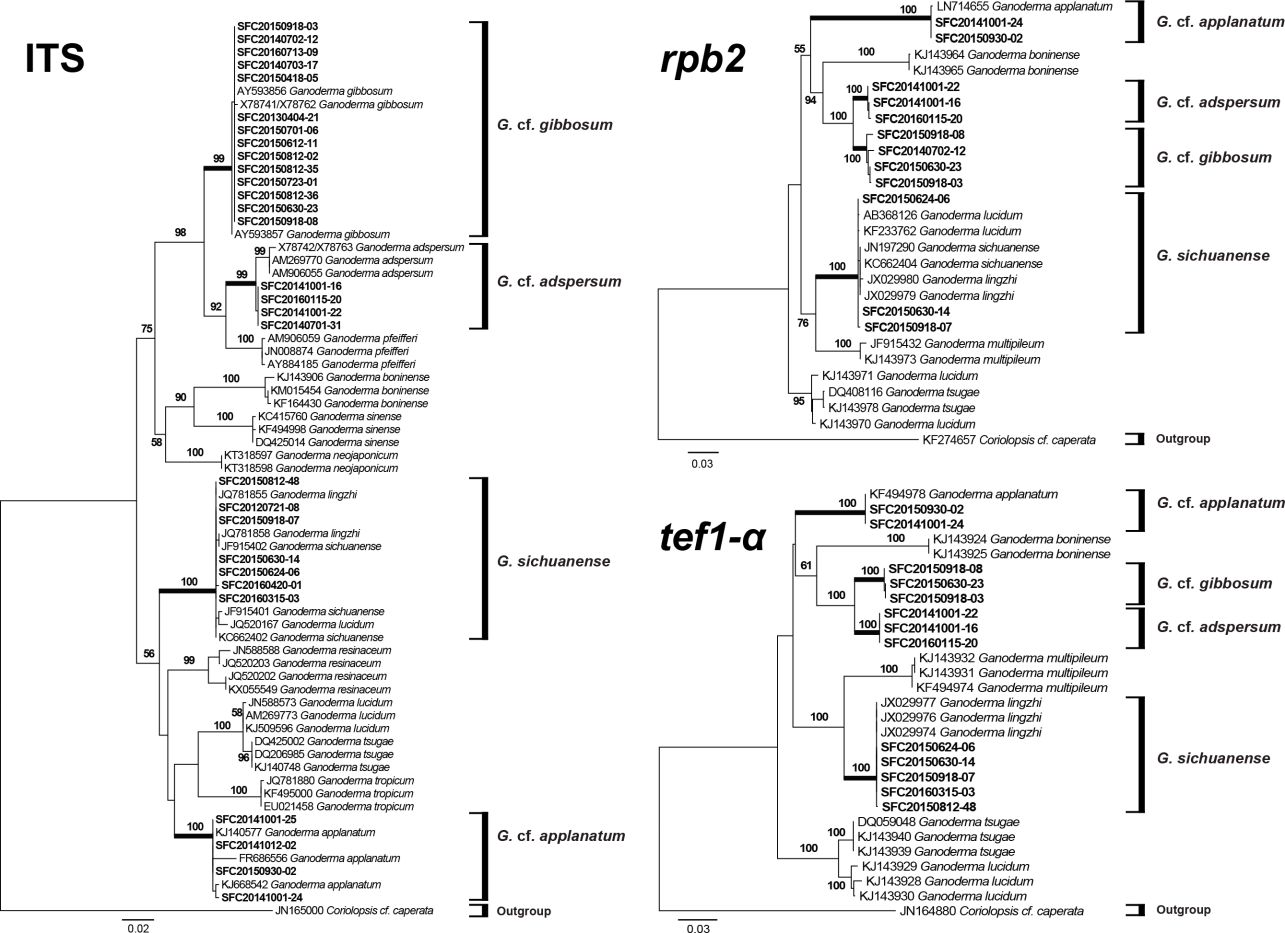
Phylogenetic tree for *Ganoderma* and related species based on a maximum likelihood (ML) analysis of the internal transcribed spacer (ITS), the second largest subunit of RNA polymerase II (*rpb2*), and translation elongation factor 1-α (*tef1-α*). ML trees were constructed with RAxML 8.0.2 using the GTRGAMMA model of evolution and 1,000 bootstrap replicates. Bootstrap scores of >50 are presented at the nodes. Branches that involved SFC sequences are in bold. The scale bar indicates the number of nucleotide substitutions per site.

The ITS region was successfully amplified and sequenced for 29 representative specimens. However, sequences for the *tef1-α* and *rpb2* regions were obtained from fewer specimens (Table 1). Phylogenetic relationships inferred from the ITS, *tef1-α*, and *rpb2* ML trees exhibited a clear distinction between the four species (Fig. 3). This phylogeny supported the identification of the laccate specimens as *G. sichuanense*. The three morphological types of non-laccate specimens clearly separated into three species. We re-named type A *Ganoderma* cf*. adspersum*, type B *Ganoderma* cf*. applanatum*, and type C *Ganoderma* cf*. gibbosum* because type specimens were not included in this study. Within the ITS, *tef1-α*, and *rpb2* phylogenies, *G.* cf*. adspersum* formed a supported clade with *G.* cf. *gibbosum*. *G.* cf*. applanatum* formed a distinct cluster with these two species to the exclusion of *G. sichuanense* (Fig. 3).

### GenBank sequence validation and distribution of four *Ganoderma* species

**Figure 4:**
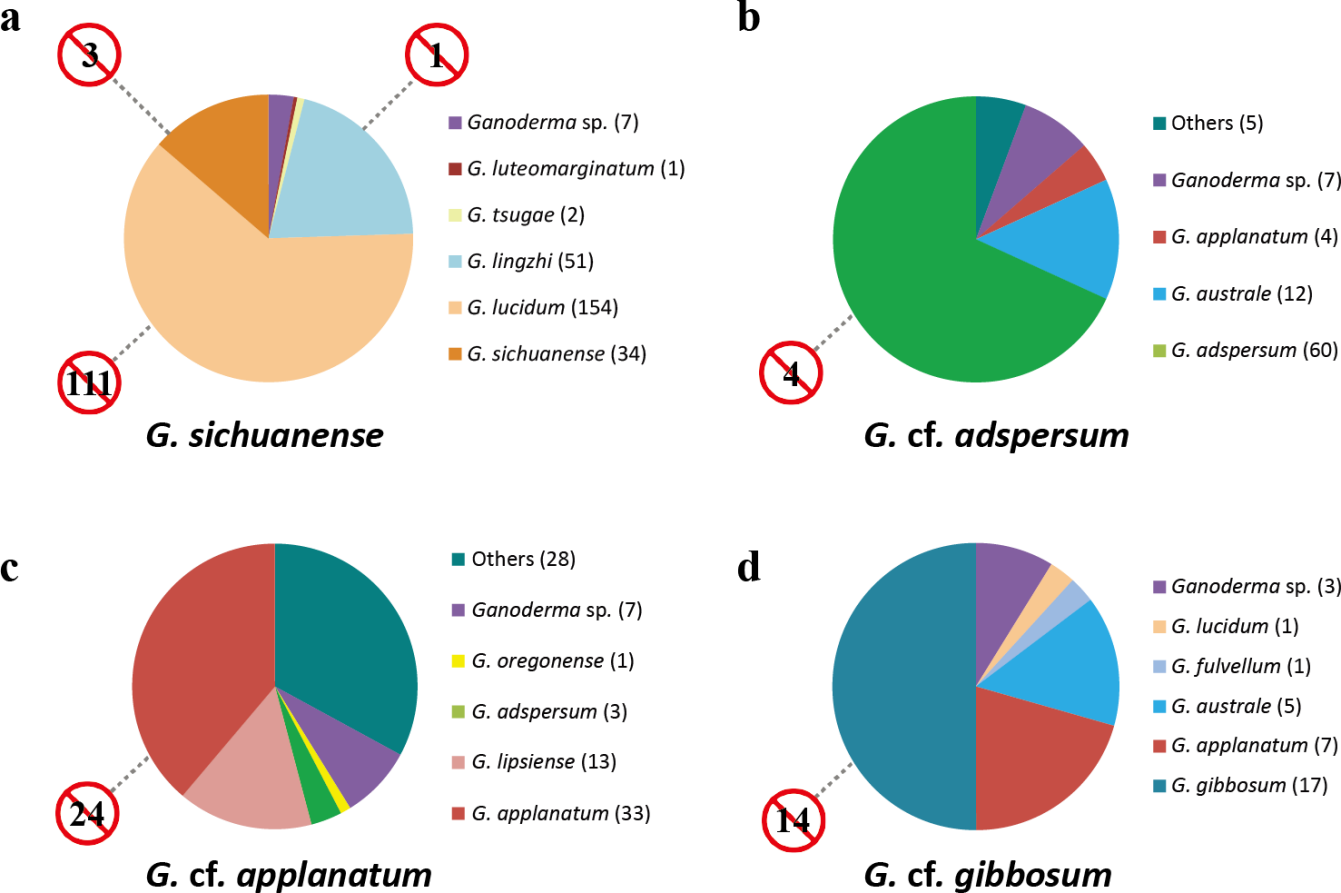
Incorrect names applied to *Ganoderma* sequences in GenBank. Color-coded taxon identifiers indicate initial GenBank annotations (number of sequences are in parentheses); “other” indicates annotations labeled as “*Ganoderma* clone” or as non-*Ganoderma* genera. Numbers in barred circles represent GenBank submissions that were incorrectly identified as indicated species.

Using BLAST searches and phylogenetic analysis, we were able to validate the sequences of four *Ganoderma* species in GenBank. Of 249 ITS sequences that were identified by this study as *G. sichuanense*, 239 were annotated in GenBank as *G. sichuanense*, *G. lucidum* or *G. lingzhi*, seven were undetermined (*Ganoderma* sp.) and three were mislabeled (two as *G. tsugae* and one as *G. luteomarginatum*). One GenBank sequence submission was incorrectly identified as *G. lingzhi* and three were misidentified as *G. sichuanense*. 111 GenBank submissions that were initially annotated in GenBank as *G. lucidum* were neither Asian (*G. sichuanense*) or European (*G. lucidum*) *Ganoderma* species. Thus, of 354 GenBank submissions labeled as *G. sichuanense* or its synonyms, 115 (32%) were found to belong to different species (Fig. 4, Table S2).

88 ITS sequences were defined by this study as *G.* cf*. adspersum*. Sixteen *G.* cf. *adspersum* sequences were mislabeled in GenBank as either *G. applanatum* (4) or *G. australe* (12). Seven were ambiguously labeled (as *Ganoderma* sp.). One sequence was labeled “*Ganoderma* clone” and four were misidentified as non-*Ganoderma* genera. In addition, we found four GenBank sequences that were erroneously labeled as *G. adspersum* (Fig. 4, Table S2).

Of 85 ITS sequences defined as *G.* cf. *applanatum* in this study, just 46 (54%) were correctly labeled as *G. applanatum* or its synonym, *G. lipsiense*. Four ITS sequences were mislabeled *Ganoderma* species (three as *G. adspersum* and one as *G. oregonense*) and seven were ambiguously labeled (e.g., *Ganoderma* sp.). Of 70 GenBank sequence submissions originally labeled as *G. applanatum* or its synonym *G. lipsiense*, 24 (34%) were found to belong to different species (Fig. 4, Table S2).

We identified 34 GenBank sequences as *G.* cf. *gibbosum*. A total of 17 GenBank sequences were correctly annotated, while 14 were initially misidentified as other *Ganoderma* species (seven as *G. applanatum*, five as *G. australe*, one as *G. fulvellum*, and one as *G. lucidum*). Three *G.* cf. *gibbosum* sequences were ambiguously labeled as “*Ganoderma* sp.”. A total of 14 of 31 (45%) GenBank sequence submissions that were initially identified as *G. gibbosum* were found to be different species (Fig. 4).

Based on the corrected database, we generated a distribution map for each species (*G. sichuanense, G.* cf*. adspersum*, *G.* cf. *applanatum*, and *G.* cf. *gibbosum*) (Fig. S1). *G. sichuanense* was distributed throughout Asia (e.g., China, Japan, Korea, Bangladesh, Malaysia, and Nepal); although nearly 70% of the *G. sichuanense* sequences were described from China. While some sequences were identified as from Poland and Italy, their specimen information lacked confirmation due to directly deposition without publication. Most *G. cf. adspersum* sequences were from European countries (Italy, Germany, Poland, United Kingdom, Austria, Finland, and France), while a small number of sequences were from Asia (India, Japan, and Korea). *G.* cf. *applanatum* sequences had a global distribution (USA, Canada, Lithuania, Hungary, Germany, Poland, Korea, and Antarctica). *G.* cf. *gibbosum* sequences were mostly limited to Asia (Korea, China, Japan, and India), with one group of sequences identified as from Poland (unpublished sequences).

## Discussion

Morphological and molecular analysis of Korean *Ganoderma* specimens collected during the last fifty years indicated that there are four *Ganoderma* species in Korea. Among the four previously described *Ganoderma* species, *G. neojaponicum* and *G. tsugae* were not found in this study. Although one specimen that was identified as *G. neojaponicum* was shown to be *G. sichuanense*, further study is needed to establish whether *G. neojaponicum* and *G. tsugae* exist in Korea.

*G. sichuanense* was previously identified and named *G. lucidum* in Korea. Recently Kwon et al. (2016) suggested that *G. lucidum* cultivated in Korea (locally known as Yeongji) was actually *G. lingzhi*, although we argue for renaming all *G. lingzhi* as *G. sichuanense* (see above). *G. sichuanense* is easily distinguished from the other three Korean *Ganoderma* species by differences in surface texture of the pileus and basidiospore size (Moncalvo & Ryvarden, 1997; Tham, 1998). Laccate pileus with longer stipe and larger basidiospores than other Asian *Ganoderma* species are typical characters of *G. sichuanense* (Wang et al., 2012). Furthermore, phylogenetic analysis confirmed that the Korean *G. sichuanense* sequences used in this study were nearly identical to the epitype for *G. sichuanense* (KC662402) (Yao, Wang & Wang, 2013).

The three species with non-laccate basidiocarps, *G.* cf. *adspersum*, *G.* cf. *applanatum*, and *G.* cf. *gibbosum*, have similar morphological characteristics which often lead to misidentification of these species, although basidiocarp morphology has been suggested to differentiate these species. Basidiocarps of *G.* cf. *adspersum* (40–100 mm) are usually thicker than those of *G.* cf. *applanatum* (20–60 mm) at the base. In addition, the undersides of the basidiocarps of *G.* cf. *adspersum* have a decurrent attachment, whereas those of *G.* cf. *applanatum* tend to emerge sharply at right angles from the host stem (Ryvarden & Gilbertson, 1993; Schwarze & Ferner, 2003). In a radial section of the hymenophore of the older parts of the fruiting body, those of *G.* cf. *adspersum* remain empty but the pores of *G.* cf. *applanatum* become filled with a white mycelium (Breitenbach & Kränzlin, 1986). *G.* cf. *adspersum* is distinguished from *G.* cf. *applanatum* by having larger basidiospores (Steyaert, 1972; Ryvarden & Gilbertson, 1993). *G.* cf. *gibbosum* is distinguished from *G.* cf. *applanatum* by the presence of the stipe (Blume & Nees von Esenbeck, 1826). It has been suggested, however, that stipe formation may be an adaptive feature because individuals of the *G. applanatum-australes* complex can develop a stipe in the tropics and stipe formation can be induced in the laboratory in strains of *G. applanatum-australes* complex species (Moncalvo & Ryvarden, 1997). Nevertheless, Korean specimens in our study were distinguished by three characteristics: The presence of the stipe discriminated *G.* cf. *gibbosum* from *G.* cf. *adspersum* and *G.* cf. *applanatum* (Fig. 1) and larger basidiospore and pore size discriminated *G.* cf. *adspersum* from *G.* cf. *applanatum* (Fig. 2).

Despite similar morphology, a multigene phylogenetic analysis showed that *G.* cf. *adspersum*, *G.* cf. *applanatum*, and *G.* cf. *gibbosum*, are distinct species (Fig. 3) corresponding to clades 2, 1, and 5, respectively, of the *Ganoderma* global phylogeny that was constructed by Moncalvo & Buchanan (2008). Our results also support previous phylogenetic reconstructions where *G.* cf. *adspersum* and *G.* cf. *applanatum* were clearly separated by rDNA analysis and further distinguished by species specific PCR primers (Gottlieb, Ferrer & Wright, 2000; Guglielmo et al., 2008). While *G.* cf. *adspersum* and *G*. cf. *gibbosum* formed a supported clade separate from *G*. *sichuanense*, *G.* cf. *adspersum* and *G. cf. gibbosum* appear to be closely related with 100% bootstrap support in *tef1-α* and *rpb2* phylogenetic trees (Fig. 3).

Our study found that the number of misidentified sequences of the four *Ganoderma* species in GenBank was substantial (Table S2, Fig. 4), with ITS sequences being significantly more likely to be misidentified than other loci due to their over-representation among phylogenetic markers. Open DNA databases (DB) such as GenBank are an important tool for species identification. In medicinal fungi, such as *Ganoderma* species, the need for satisfactory taxonomic sampling and accurate identification in DBs is critical. Among the four species, the highest number of ITS sequences listed on GenBank was those of *G. sichuanense* (Fig. 4) and the unusually high number of *G. sichuanense* sequences found on GenBank is likely due to the economic and medicinal importance of the species. In order to minimize confusion and misidentification in future studies, we strongly recommend that the names *G. lucidum* and *G. lingzhi* be avoided and all new submissions of this species be labeled *G. sichuanense.* The distribution of *G. sichuanense* appears to be limited to Asia, with specimens reported from China, Korea, Japan, Bangladesh, Malaysia, and Nepal (Fig. S1).

Our study shows that *G.* cf. *adspersum* sequences in GenBank were commonly misidentified as *G. australe* (Fig. 4). Based on morphological analysis, *G.* cf. *adspersum* was considered a synonym of *G. australe* by Ryvarden (1976) and Ryvarden & Gilbertson (1993); however, Smith & Sivasithamparam (2000), using ITS sequence data, argued that *G. adspersum* and *G. australe* are two distinct species. *G. adspersum* was commonly reported from Europe, where type specimens were collected (Moncalvo & Ryvarden, 1997; Smith & Sivasithamparam, 2000). Our analysis confirmed that *G.* cf. *adspersum* occurs in Europe, but is distributed in Asia and North America as well (Fig. S1). Erroneously annotated sequences were also common among *G.* cf. *applanatum* GenBank submissions. 34% of sequences that were initially annotated as *G.* cf. *applanatum* were shown to be other species while just 54% of authentic *G.* cf. *applanatum* sequences were initially annotated as such in GenBank. A total of 13 sequences that were labeled as *G. lipsiense* in GenBank were included in the *G.* cf. *applanatum* clade because *G. lipsiense* is synonymous with *G.* cf. *applanatum* (Moncalvo & Ryvarden, 1997). Our analysis confirmed that *G.* cf. *applanatum* has a global distribution (Moncalvo & Ryvarden, 1997) with sequences reported from Europe, Asia and North America (Fig. S1). Nearly half of all GenBank sequences annotated as *G.* cf. *gibbosum* were misidentified and the same proportion of authentic *G.* cf. *gibbosum* sequences were initially annotated as different species. *G.* cf. *gibbosum* has a primarily Asian distribution, and the ecto-type was initially reported from Java, Indonesia (Blume & Nees von Esenbeck, 1826), although scattered samples were reported from Eastern Europe.

In conclusion, as we constructed phylogenetic trees using reference sequences from GenBank, it became apparent that many *Ganoderma* reference sequences were misidentified. In this study, we identified incorrectly labeled sequences on GenBank and constructed new phylogenies with reference sequences that were correctly assigned to specific taxa. This study will provide a framework for future efforts to replace inaccurate public information with reliable taxonomic assignments. We strongly encourage the authors of previously submitted specimens that have been shown to be misidentified or use inappropriate species names (i.e., *lucidum* and *lingzhi*) to correct these submissions on GenBank. This improvement is vital not only for fungal taxonomists, but given the diverse ecological, medicinal, and economic impacts of *Ganoderma* species, this project will be of value to researchers across multiple disciplines.

##  Supplemental Information

10.7717/peerj.3596/supp-1 Figure S1 Supplementary Figure 1

10.7717/peerj.3596/supp-2 Table S1 Supplementary Table 1

10.7717/peerj.3596/supp-3 Table S2 Supplementary Table 2

## Additional Information and Declarations

### Data Availability

The following information was supplied regarding data availability: The raw data (DNA sequences) are available in GenBank: KY364244 –KY364272.
