# Supplementary material for: Taxonomic evaluation of selected Ganoderma species and database sequence validation
Source: PeerJ. 2017 Jul 27;5:e3596. doi: 10.7717/peerj.3596 (PMC5534161; doi:10.7717/peerj.3596)
Supplement: Figure S1 [file peerj-05-3596-s001.pdf]

**Fig. S1** Geographic distribution of four *Ganoderma* species based on the validated sequence database generated by this study. Blue circles represent location of specimen collection from published papers. Gray circles represent location of direct GenBank submission. SFC sequences were not included in the map.

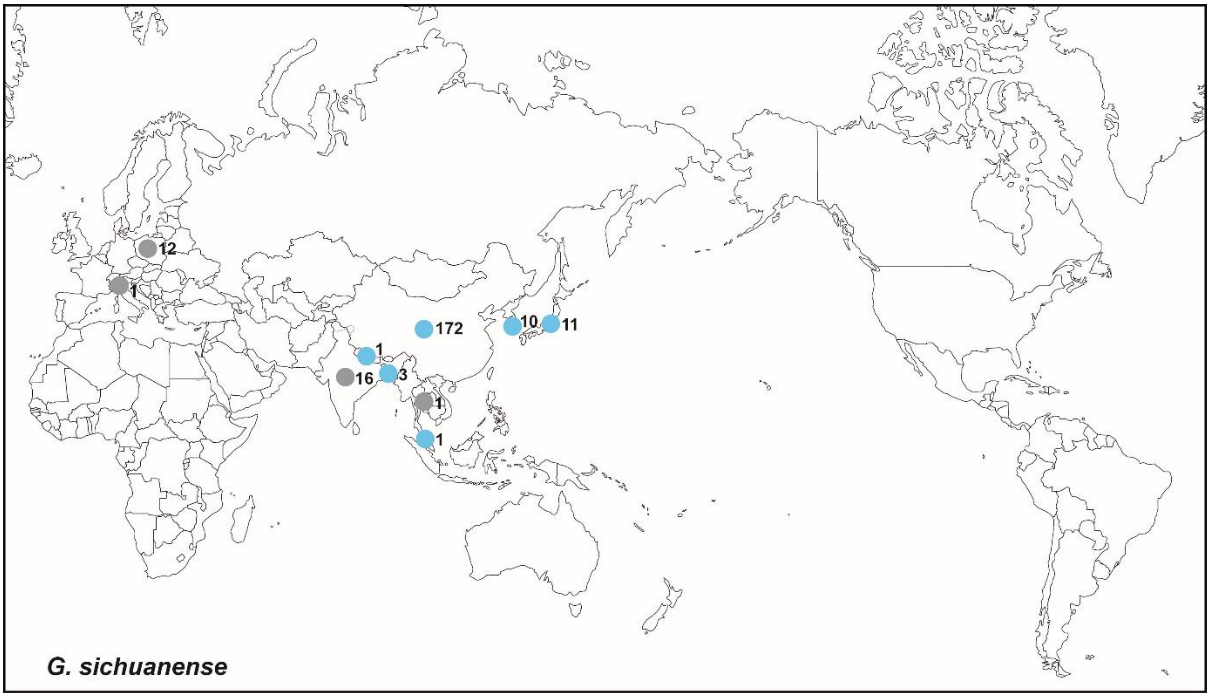


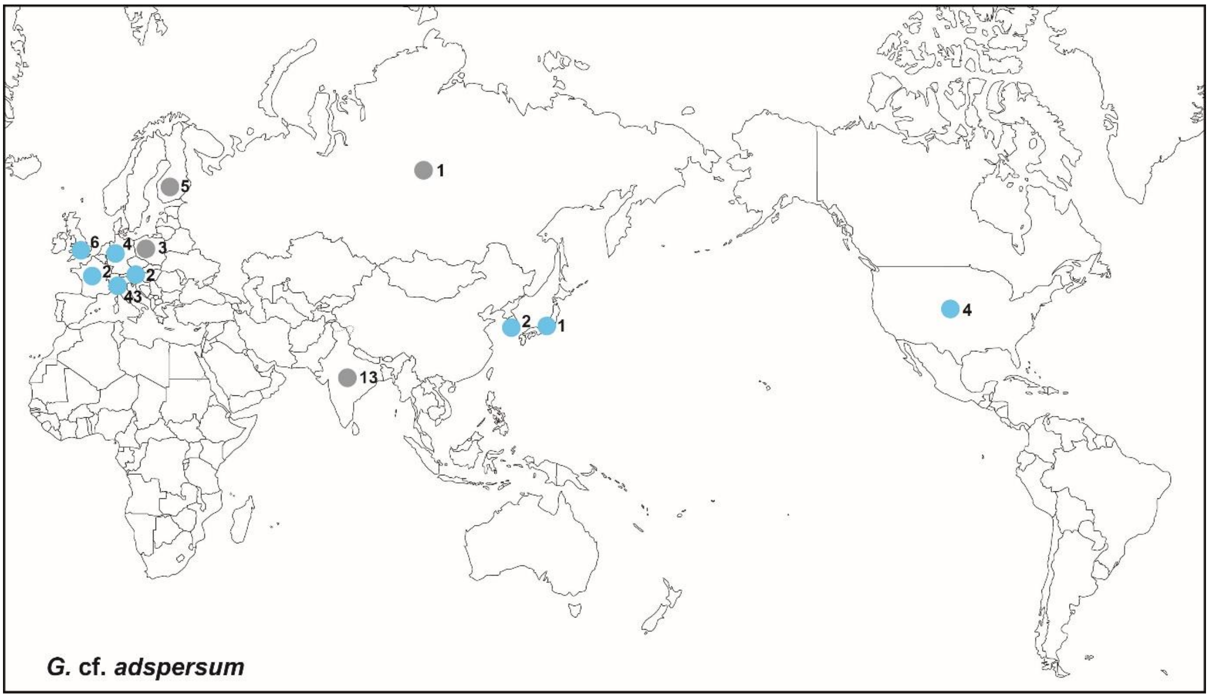

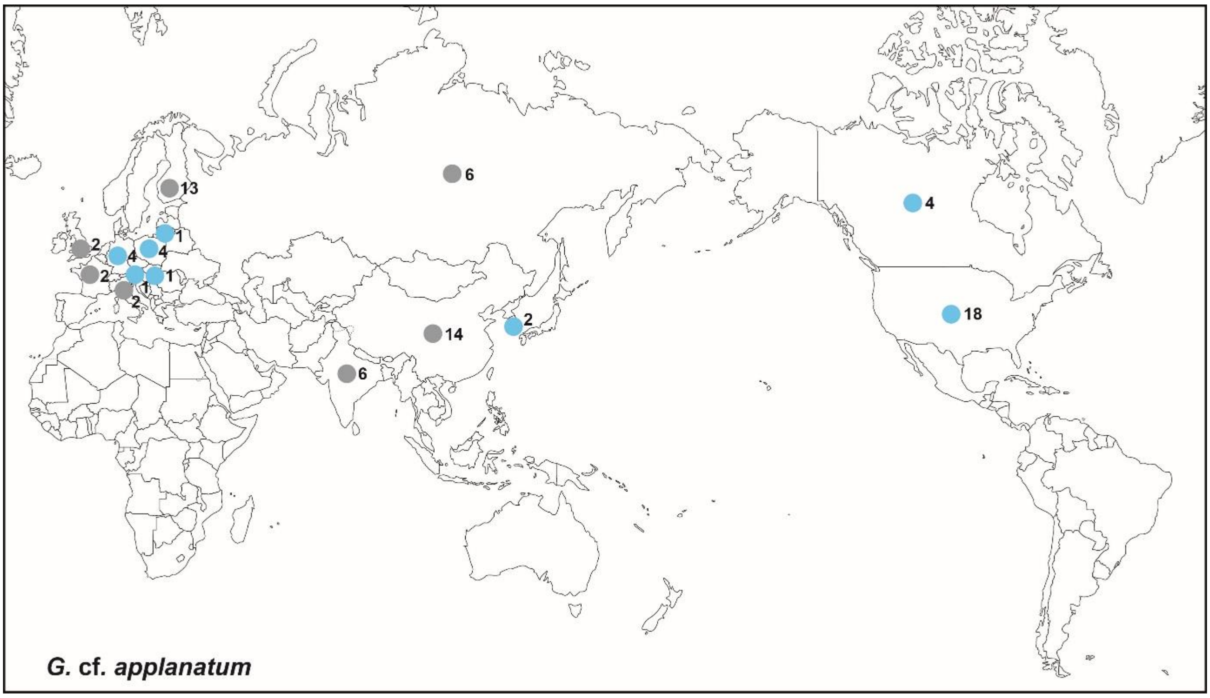
6
13
2
4
4
1
2
1
1
2
14
6
2
4
18
G. cf. applanatum

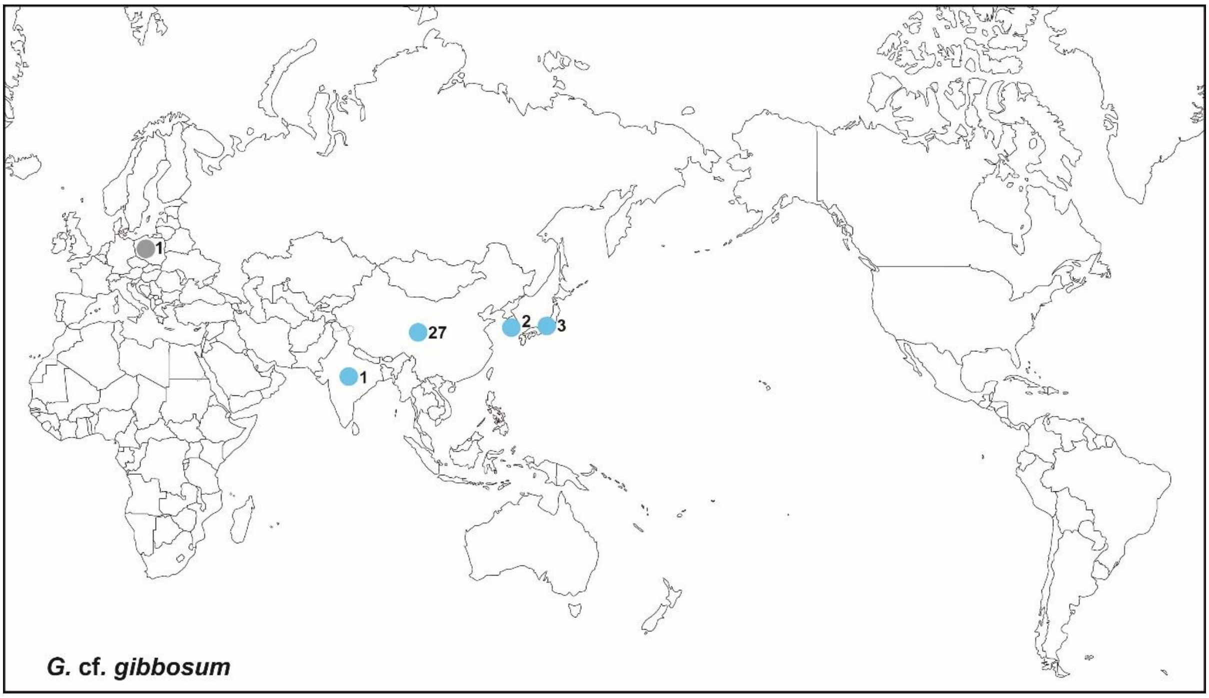
1
27
2
3
1
G. cf. gibbosum
