# Supplementary material for: Taxonomic evaluation of selected Ganoderma species and database sequence validation
Source: PeerJ. 2017 Jul 27;5:e3596. doi: 10.7717/peerj.3596 (PMC5534161; doi:10.7717/peerj.3596)
Supplement: Table S1 [file peerj-05-3596-s002.docx]

**Table S1** *Ganoderma* specimens from the Seoul National University Fungus Collection (SFC) and Korea Mushroom Resource Bank (KMRB) used in this study

| **Species** | **Specimen No.** | **Locality** | **Pore no.** | **Basidiospore size** | **Stipe**^c^ |
| --- | --- | --- | --- | --- | --- |
| *G. sichuanense* | SFC19690921-01 | -^a^ | 6 | 10.4-12.2 × 6.4-7.3 | o |
|  | SFC19691019-03 | Jikdong-ri, Sohol-eup, Pocheon-si, Gyeonggi-do, Korea | 7 | 9.8-11.0 × 6.4-7.1 | o |
|  | SFC19691019-04 | Jikdong-ri, Sohol-eup, Pocheon-si, Gyeonggi-do, Korea | 6-7 | ND^b^ | o |
|  | SFC19710726-18 | Waryong-dong, Jongno-gu, Seoul, Korea | 6 | 11.4-12.0 × 6.7-7.3 | o |
|  | SFC19930706-01 | Osaeng-ri, Saenggeuk-myeon, Eumseong-gun, Chungcheongbuk-do, Korea | 6 | ND | o |
|  | SFC19960704-01 | 1-dong Samseong, Gangnam-gu, Seoul, Korea | 6 | 10.4-11.0 × 6.7-7.0 | o |
|  | SFC19980708-04 | Cheongnyangri-dong, Dongdaemun-gu, Seoul, Korea | 6 | 11.5-12.2 × 6.4-7.3 | o |
|  | SFC19980708-07 | Cheongnyangri-dong, Dongdaemun-gu, Seoul, Korea | 6 | 11.4-11.5 × 6.9-7.2 | o |
|  | SFC19990123-17 | Bugeun-ri, Hajeom-myeon, Ganghwa-gun, Incheon, Korea | 6 | 10.4-12.2 × 6.9-7.2 | o |
|  | SFC19990729-56 | Okdang-ri, Gwansan-eup, Jangheung-gun, Jeollanam-do, Korea | 6 | 10.4-12.2 × 6.4-7.1 | o |
|  | SFC19990924-10 | Naega-myeon, Ganghwa-gun, Incheon, Korea | 5-6 | 10.8-11.4 × 7.0-7.3 | o |
|  | SFC19990924-15 | Naega-myeon, Ganghwa-gun, Incheon, Korea | 5-7 | 9.9-12.0 × 6.8-7.0 | o |
|  | SFC19991218-09 | Jeokseong-ri, Dongno-myeon, Mungyeong-si, Gyeongsangbuk-do, Korea | 6 | 9.8-12.2 × 6.6-7.1 | o |
|  | SFC20010316-14 | Onsu-ri, Gilsang-myeon, Ganghwa-gun, Incheon, Korea | 6-7 | 10.5-11.6 × 6.6-7.2 | o |
|  | SFC20021109-08 | Songnisan-myeon, Boeun-gun, Chungcheongbuk-do, Korea | 6-7 | 10.9-11.5 × 6.7-6.9 | o |
|  | SFC20030828-108 | Sojeong-ri, Buksang-myeon, Geochang-gun, Gyeongsangnam-do, Korea | 6 | 9.9-10.8 × 6.4-7.4 | o |
|  | SFC20041028-01 | Oeyeondo-ri, Ocheon-myeon, Boryeong-si, Chungcheongnam-do, Korea | 6 | 10.5-11.1 × 6.6-7.4 | o |
|  | SFC20050609-06 | Songjeong-ri, Mijo-myeon, Namhae-gun, Gyeongsangnam-do, Korea | 6 | 10.4-12.2 × 6.4-7.4 | o |
|  | SFC20050721-03 | Songjeong-ri, Mijo-myeon, Namhae-gun, Gyeongsangnam-do, Korea | 6-7 | 11.0-12.2 × 7.0-7.3 | o |
|  | SFC20050802-32 | Hansan island, Tongyeong-si, Gyeongsangnam-do, Korea | 7 | 10.1-11.2 × 6.6-6.9 | o |
|  | SFC20050827-54 | SInje-gunon-ri, Sanyang-eup, Tongyeong-si, Gyeongsangnam-do, Korea | 6 | ND | o |
|  | SFC20050831-09 | Geoje island, Gyeongsangnam-do, Korea | 6 | 10.5-11.6 × 6.6-7.2 | o |
|  | SFC20050831-106 | Geoje island, Gyeongsangnam-do, Korea | 6 | 11.5-12.2 × 6.4-7.4 | o |
|  | SFC20050831-34 | Geoje island, Gyeongsangnam-do, Korea | 6 | 10.0-11.3 × 6.7-7.2 | o |
|  | SFC20050831-42 | Geoje island, Gyeongsangnam-do, Korea | 6 | 11.4-12.2 × 6.5-7.1 | o |
|  | SFC20110709-15 | Jeongbalsan-dong Ilsandong-gu, Goyang-si, Gyeonggi-do, Korea | 6 | 10.9-11.6 × 6.9-7.3 | o |
|  | SFC20110823-13 | Sillim-dong, Gwanak-gu, Seoul, Korea | 6 | 10.3-11.0 × 6.4-7.1 | o |
|  | SFC20120721-08 | Singok-ri, Gochon-eup, Gimpo-si, Gyeonggi-do, Korea | 6 | 10.5-11.6 × 6.6-7.2 | o |
|  | SFC20120804-12 | Mangwol-ri, Hajeom-myeon, Ganghwa-gun, Incheon, Korea | 6 | 10.1-11.2 × 6.6-7.3 | o |
|  | SFC20120817-22 | Sillim-dong, Gwanak-gu, Seoul, Korea | 6 | 10.4-12.2 × 6.8-7.1 | o |
|  | SFC20120820-25 | Daegok-ri, Haemi-myeon, Seosan-si, Chungcheongnam-do, Korea | 6 | 10.9-11.5 × 6.4-7.3 | o |
|  | SFC20130506-12 | Urae-ri, Bomun-myeon, Yecheon-gun, Gyeongsangbuk-do, Korea | 6 | 10.0-11.3 × 6.7-7.2 | o |
|  | SFC20140819-13 | Hakgok-ri, Socho-myeon, Wonju-si, Gangwon-do, Korea | 6 | 11.0-12.2 × 6.5-7.1 | o |
|  | SFC20150407-04 | Hwado-myeon, Ganghwa-gun, Incheon, Korea | 6 | 10.4-12.2 × 6.4-7.1 | o |
|  | SFC20150612-10 | Samhwa-dong, Donghae-si, Gangwon-do, Korea | 6 | 10.4-12.2 × 7.0-7.2 | o |
|  | SFC20150624-06  (KMRB 15062406) | Gigye-myeon, Buk-gu, Pohang-si, Gyeongsangbuk-do, Korea | 6 | 11.4-12.2 × 6.5-7.1 | o |
|  | SFC20150630-14  (KMRB 15063014) | Hunjeong-dong, Jongno-gu, Seoul, Korea | 6-7 | 10.4-12.2 × 6.4-7.0 | o |
|  | SFC20150715-19 | Deokyang-gu, Goyang-si, Gyeonggi-do, Korea | 6 | 11.4-11.9 × 6.7-7.3 | o |
|  | SFC20150716-14 | Hwado-myeon, Ganghwa-gun, Incheon, Korea | 6 | 10.9-12.0 × 6.4-7.0 | o |
|  | SFC20150723-10 | Jongmyo, 157 Jong-ro, Jongno-gu, Seoul, Korea | 6 | ND | o |
|  | SFC20150812-48 | Jongmyo, 157 Jong-ro, Jongno-gu, Seoul, Korea | 6 | ND | o |
|  | SFC20150818-42 | Heungwang-ri, Hwado-myeon, Ganghwa-gun, Incheon, Korea | 6 | 11.7-12.1 × 6.4-7.0 | o |
|  | SFC20150908-31 | Heungwang-ri, Hwado-myeon, Ganghwa-gun, Incheon, Korea | 6 | 11.4-12.0 × 6.5-7.4 | o |
|  | SFC20150917-12 | 197 Donggureung-ro, Guri-si, Gyeonggi-do, Korea | 6 | 10.1-11.2 × 6.6-7.3 | o |
|  | SFC20150918-07 | Jongmyo, 157 Jong-ro, Jongno-gu, Seoul, Korea | 5-6 | 10.1-11.2 × 6.5-7.2 | o |
|  | SFC20151029-08 | Jikdong-ri, Sohol-eup, Pocheon-si, Gyeonggi-do, Korea | 6 | 10.4-12.2 × 6.4-7.4 | o |
|  | SFC20151230-01 | Dusan-ri, Namil-myeon, Cheongwon-gun, Chungcheongbuk-do, Korea | 5-6 | 11.0-11.3 × 6.8-7.1 | o |
|  | SFC20160420-01 | - | 6 | 10.0-10.4 × 6.6-7.4 | o |
|  | SFC20160712-26 | Bukdo-myeon, Ongjin-gun, Incheon Korea | 7 | 10.2-12.1 × 6.4-7.4 | o |
|  | SFC20160819-08 | Yongdu-dong, Deokyang-gu, Goyang-si, Gyeonggi-do Korea | 7 | 10.4-11.0 × 6.8-7.4 | o |
| *G.* cf. *adspersum* | SFC20140701-31  (KMRB 14070131) | Buk-myeon, Inje-gun, Gangwon-do, Korea | 3 | 9.1-9.7 × 6.4-6.9 | x |
|  | SFC20141001-16 | Girin-myeon, Inje-gun, Gangwon-do, Korea | 4 | 9.7-10.1 × 5.9-6.8 | x |
|  | SFC20141001-22 | Girin-myeon, Inje-gun, Gangwon-do, Korea | 4 | 10.1-11.3 × 6.9-7.8 | x |
|  | SFC20160115-20  (KMRB 16011520) | SInje-gunom-ri, Yongmun-myeon, Yangpyeong-gun, Gyeonggi-do, Korea | 4 | 7.8-9.9 × 5.1-7.0 | x |
| *G.* cf. *applanatum* | SFC19950527-13 | Sangui-ri Budong-myeon, Cheongsong Gyeongsangbuk-do, Korea | 6 | 8.7-8.9 × 5.8-5.9 | x |
|  | SFC19960706-09 | Sucheol-ri, Punggi-eup, Yeongju, Gyeongsangbuk-do, Korea | 6 | 8.5-8.7 × 5.4-5.9 | x |
|  | SFC19960921-09 | Baekdun-ri, Buk-myeon, Gapyeong-gun, Gyeonggi-do, Korea | 5-6 | 8.1-8.5 × 5.6-5.7 | x |
|  | SFC19981217-42 | Songgye-ri, Hansu-myeon, Jecheon-si, Chungcheongbuk-do, Korea | ND | ND | x |
|  | SFC19990709-25 | Yongdae-ri, Buk-myeon, Inje-gun, Gangwon-do, Korea | 6 | 8.5-8.7 × 5.4-5.9 | x |
|  | SFC19990729-51 | Okdang-ri, Gwansan-eup, Jangheung-gun, Jeollanam-do, Korea | 5-6 | 8.1-8.6 × 5.5-6.3 | x |
|  | SFC19990914-25 | Bangdong-ri, Girin-myeon, Inje-gun, Gangwon-do, Korea | 6-7 | 8.3-8.4 × 5.4-5.9 | x |
|  | SFC20011115-03 | Hwagae-myeon, Hadong-gun, Gyeongsangnam-do, Korea | 6 | 8.1-8.6 × 5.5-6.3 | x |
|  | SFC20011115-04 | Hwagae-myeon, Hadong-gun, Gyeongsangnam-do, Korea | 6 | ND | x |
|  | SFC20011115-12 | Hwagae-myeon, Hadong-gun, Korea | ND | ND | x |
|  | SFC20020418-20 | Songnisan-myeon, Boeun-gun, Chungcheongbuk-do, Korea | ND | ND | x |
|  | SFC20030613-32 | Samgong-ri, Seolcheon-myeon, Muju, Jeollabuk-do, Korea | 5-6 | 8.2-8.6 × 5.7-5.9 | x |
|  | SFC20031006-54 | Jukcheon-ri, Anseong-myeon, Muju, Jeollabuk-do, Korea | 5-6 | ND | x |
|  | SFC20040103-11 | Hyeol-dong, Taebaek-si, Gangwon-do, Korea | 6-7 | ND | x |
|  | SFC20040526-20 | Hwagae-myeon, Hadong-gun, Gyeongsangnam-do, Korea | 5-6 | ND | x |
|  | SFC20060809-09 | Cheondong-ri, Danyang-eup, Danyang-gun, Chungcheongbuk-do, Korea | 6 | 8.2-8.6 × 5.7-5.9 | x |
|  | SFC20120704-20 | Yongdae-ri, Buk-myeon, Inje-gun, Gangwon-do, Korea | 5-6 | 8.1-8.6 × 5.5-6.3 | x |
|  | SFC20130427-08 | Sodo-dong, Taebaek-si, Gangwon-do, Korea | 6 | 8.1-8.3 × 5.8-6.0 | x |
|  | SFC20130521-51 | Sodo-dong, Taebaek-si, Gangwon-do, Korea | 6 | 8.3-8.9 × 5.3-5.4 | x |
|  | SFC20140701-35 | Yongdae-ri, Buk-myeon, Inje-gun, Gangwon-do, Korea | 6-7 | ND | x |
|  | SFC20141001-24 | Girin-myeon, Inje-gun, Gangwon-do, Korea | 6-7 | 8.0-8.7 × 5.4-5.9 | x |
|  | SFC20141001-25 | Inje-gun, Gangwon-do, Korea | 5-6 | 8.1-8.8 × 5.6-5.9 | x |
|  | SFC20141001-26 | Inje-gun, Gangwon-do, Korea | 6-7 | 8.3-8.5 × 5.5-5.9 | x |
|  | SFC20141012-02 | Jindong-ri, Girin-myeon, Inje-gun, Gangwon-do, Korea | 6-7 | ND | x |
|  | SFC20150319-19 | Jindong-ri, Girin-myeon, Inje-gun, Gangwon-do, Korea | 6 | 8.0-8.7 × 5.4-5.9 | x |
|  | SFC20150701-84 | Jochon-eup, Jeju-si, Jeju-do, Korea | 6 | ND | x |
|  | SFC20150718-09 | Jinbu-myeon, Pyeongchang-gun, Gangwon-do, Korea | 5-6 | 8.3-8.4 × 5.4-5.9 | x |
|  | SFC20150721-14 | Jindong-ri, Girin-myeon, Inje-gun, Gangwon-do, Korea | 6-7 | ND | x |
|  | SFC20150902-22 | Nari, Buk-myeon, Ulleung-gun, Gyeongsangbuk-do, Korea | 6 | 8.3-8.5 × 5.5-5.9 | x |
|  | SFC20150930-02  (KMRB 15093002) | Buk-myeon, Inje-gun, Gangwon-do, Korea | 6-7 | 8.1-8.8 × 5.6-5.8 | x |
| *G.* cf. *gibbosum* | SFC19960806-01 | Yesong-ri, Bogil-myeon, Wando, Jeollanam-do, Korea | 5 | ND | o |
|  | SFC19961111-28 | Mungyeong-si-eup, Mungyeong-si, Gyeongsangbuk-do, Korea | ND | ND | x |
|  | SFC19991218-09 | Jeokseong-ri, Dongno-myeon, Mungyeong-si, Gyeongsangbuk-do, Korea | 5 | 7.7-8.0 × 4.9-5.1 | o |
|  | SFC20011222-07 | Hakbong-ri, Banpo-myeon, Gongju, Chungcheongnam-do, Korea | 4 | 7.7-7.9 × 4.9-5.4 | o |
|  | SFC20020620-15 | Songnisan-myeon, Boeun-gun, Chungcheongbuk-do, Korea | 4-5 | 9.1-9.4 × 5.5-6.3 | x |
|  | SFC20041029-02 | Oeyeondo-ri, Ocheon-myeon, Boryeong-si, Chungcheongnam-do, Korea | 5 | 8.7-9.3 × 5.3-5.8 | x |
|  | SFC20060525-18 | Samgong-ri, Seolcheon-myeon, Muju, Jeollabuk-do, Korea | 5 | 7.8-8.3 × 5.9-6.3 | x |
|  | SFC20120814-27 | Nari, Buk-myeon, Ulleung-gun, Gyeongsangbuk-do, Korea | 5 | ND | x |
|  | SFC20130404-21 | Namgok-ri, Euncheok-myeon, Sangju, Gyeongsangbuk-do, Korea | 5 | ND | o |
|  | SFC20130809-03 | Namgok-ri, Euncheok-myeon, Sangju, Gyeongsangbuk-do, Korea | 5 | 8.1-8.3 × 5.5-6.3 | o |
|  | SFC20140701-30 | Jindong-ri, Girin-myeon, Inje-gun, Gangwon-do, Korea | 4-5 | 8.1-8.3 × 5.5-6.3 | x |
|  | SFC20140702-12 | Gamsan-ri, Andeok-myeon, Seogwipo, Jeju-do, Korea | 5 | 8.1-8.6 × 5.5-6.3 | o |
|  | SFC20140703-17 | Bongae-dong, Jeju-si, Jeju-do, Korea | 5 | ND | o |
|  | SFC20141001-21 | Girin-myeon, Inje-gun, Gangwon-do, Korea | 4 | 7.9-8.3 × 4.9-5.2 | x |
|  | SFC20150418-05 | Shillim-dong, Gwanak-gu, Seoul, Korea | 5 | 7.9-8.7 × 5.0-5.9 | o |
|  | SFC20150612-11  (KMRB 15061211) | Samhwa-dong, Donghae-si, Gangwon-do, Korea | ND | ND | o |
|  | SFC20150630-08 | Hunjeong-dong, Jongno-gu, Seoul, Korea | 4-5 | ND | x |
|  | SFC20150630-23  (KMRB 15063023) | Hunjeong-dong, Jongno-gu, Seoul, Korea | 4-5 | ND | o |
|  | SFC20150701-06  (KMRB 15070106) | Jochon-eup, Jeju-si, Jeju-do, Korea | 5 | ND | o |
|  | SFC20150723-01  (KMRB 15072301) | Jongmyo, 157 Jong-ro, Jongno-gu, Seoul, Korea | 4 | 8.0-8.6 × 5.5-6.3 | o |
|  | SFC20150812-02  (KMRB 15081202) | Jongmyo, 157 Jong-ro, Jongno-gu, Seoul, Korea | 4-5 | 8.4-9.3 × 5.5-5.9 | o |
|  | SFC20150812-35  (KMRB 15081235) | Jongmyo, 157 Jong-ro, Jongno-gu, Seoul, Korea | 5 | 7.9-8.8 × 5.0-5.9 | o |
|  | SFC20150812-36  (KMRB 15081236) | Jongmyo, 157 Jong-ro, Jongno-gu, Seoul, Korea | 5 | 8.0-8.3 × 5.5-6.3 | o |
|  | SFC20150812-51 | Jongmyo, 157 Jong-ro, Jongno-gu, Seoul, Korea | 5 | 8.5-9.0 × 5.0-5.5 | x |
|  | SFC20150918-01 | Jongmyo, 157 Jong-ro, Jongno-gu, Seoul, Korea | 5 | 7.7-8.4 × 5.2-5.9 | o |
|  | SFC20150918-03  (KMRB 15091803) | Jongmyo, 157 Jong-ro, Jongno-gu, Seoul, Korea | 5 | 7.9-8.0 × 4.9-5.8 | o |
|  | SFC20150918-08  (KMRB 15091808) | Jongmyo, 157 Jong-ro, Jongno-gu, Seoul, Korea | 5 | 7.7-8.9 × 4.9-5.9 | o |
|  | SFC20151013-01 | Dongcheon-dong, Gyeongju, Gyeongsangbuk-do, Korea | 5 | 9.1-9.3 × 5.5-6.3 | x |
|  | SFC20160713-09 | Seonheul-ri, Jochon-eup, Jeju-si, Jeju-do, Korea | 5 | 8.4-9.3 × 5.5-5.9 | o |

^a^-: no information; ^b^ND: Not determined; ^c^: o (presence), x (absence); *G. sichuanense* specimens were initially identified as *G. lucidum* except to SFC20151029-08 (*G. neojaponicum*). *G.* cf. *adspersum*, *G.* cf. *applanatum*, and *G.* cf. *gibbosum* specimens were initially identified as *G. applanatum*.
