# Supplementary material for: Taxonomic evaluation of selected Ganoderma species and database sequence validation
Source: PeerJ. 2017 Jul 27;5:e3596. doi: 10.7717/peerj.3596 (PMC5534161; doi:10.7717/peerj.3596)
Supplement: Table S2 [file peerj-05-3596-s003.docx]

**Table S2** List of validated and incorrect sequences four *Ganoderma* species

| **Correct name** | **Labeled name** | **Accession number** |
| --- | --- | --- |
| ***G. sichuanense*** | *G. sichuanense* | \| JF915393 \| JF915394 \| JF915395 \| JF915396 \| JF915397 \| F915398 \| JF915399 \| JF915400 \| JF915401 \|  \| \| --- \| --- \| --- \| --- \| --- \| --- \| --- \| --- \| --- \| --- \| \| JF915402 \| JF915403 \| JF915404 \| JF915405 \| JF915406 \| JF915407 \| JF915408 \| JN197281 \| JN197282 \| \| JN197283 \| JN197284 \| KC505544 \| KC662402 \| KT318600 \| KT318601 \| KT318602 \| KT318603 \| KT693248 \| \| KT693249 \| KT693250 \| KT693251 \| KT693252 \| KT693253 \| KT693254 \| KT693255 \| |
|  |  | \| KX055552 \| JQ781878 \| JQ781877 \|  \|  \| \| --- \| --- \| --- \| --- \| --- \| |
|  | *G. lucidum* | \| AB462322 \| AB733122 \| AB733174 \| AB733175 \| AF506371 \| DQ424969 \| DQ424970 \| DQ424971 \| DQ424973 \| \| --- \| --- \| --- \| --- \| --- \| --- \| --- \| --- \| --- \| \| DQ424974 \| DQ424979 \| DQ424980 \| DQ424981 \| DQ424983 \| DQ424984 \| DQ424985 \| DQ424986 \| DQ424987 \| \| DQ424988 \| DQ424989 \| DQ424991 \| DQ424992 \| DQ424993 \| DQ424994 \| DQ424997 \| DQ425007 \| DQ425008 \| \| DQ425012 \| DQ425013 \| DQ425015 \| EF188277 \| EF188278 \| EF188279 \| EF188280 \| EU021455 \| EU021456 \| \| EU498091 \| EU520137 \| EU520155 \| EU520235 \| EU520247 \| FJ379262 \| FJ379263 \| FJ379265 \| FJ463904 \| \| FJ463907 \| FJ463909 \| FJ463910 \| FJ463912 \| FJ463919 \| FJ463921 \| FJ463929 \| FJ463931 \| FJ501553 \| \| FJ501554 \| FJ501555 \| FJ501557 \| FJ501559 \| FJ501568 \| FJ501569 \| FJ501576 \| FJ501577 \| FJ687271 \| \| FJ940919 \| GU213471 \| GU213476 \| GU213477 \| GU213478 \| GU213479 \| GU213480 \| GU213481 \| GU213483 \| \| GU213484 \| GU213485 \| GU213487 \| HQ222603 \| HQ222604 \| HQ235630 \| HQ235631 \| HQ235632 \| JN008869 \| \| JN008870 \| JN008871 \| JN008872 \| JN048774 \| JN222405 \| JN222421 \| JN222423 \| JN222424 \| JN222425 \| \| JN222426 \| JQ520167 \| JQ520168 \| JQ520169 \| JQ520170 \| JQ520171 \| JQ520172 \| JQ520173 \| JQ520174 \| \| JQ520175 \| JQ520176 \| JQ520177 \| JQ520178 \| JQ520179 \| JQ520180 \| JQ520181 \| JQ520182 \| JQ520183 \| \| JQ520184 \| JQ520188 \| JQ520189 \| JQ627589 \| JQ627590 \| JX162754 \| JX162755 \| JX162756 \| JX162757 \| \| JX162758 \| JX162759 \| JX162760 \| JX162761 \| JX162762 \| JX162763 \| JX162764 \| JX162765 \| JX162766 \| \| JX162767 \| JX162768 \| JX162770 \| KC311368 \| KC311370 \| KC311371 \| KC414242 \| KF146177 \| KM269294 \| \| KT185665 \| KT717953 \| KT717954 \| KT717955 \| KT717956 \| KT906367 \| KT906368 \| KT921216 \| KX055533 \| \| KX055535 \| KX055538 \| KX055539 \| KX055543 \| KX055544 \| KX055545 \| KX055550 \| KX055551 \| KX055553 \| \| LC068794 \|  \|  \|  \|  \| |
|  |  | \| AB509616 \| AF079584 \| AF506372 \| AF506373 \| AM269772 \| AY636058 \| AY636059 \| AY636068 \| AY870653 \| \| --- \| --- \| --- \| --- \| --- \| --- \| --- \| --- \| --- \| \| DQ424975 \| DQ424976 \| DQ424998 \| DQ424999 \| DQ425000 \| EU021459 \| EU021460 \| EU021461 \| EU021462 \| \| FJ263454 \| FJ463905 \| FJ463906 \| FJ463908 \| FJ463913 \| FJ463915 \| FJ463917 \| FJ463920 \| FJ463923 \| \| FJ463924 \| FJ463925 \| FJ463926 \| FJ501561 \| FJ655477 \| FJ982798 \| GQ249880 \| GQ249884 \| GQ249885 \| \| GQ249886 \| GU213482 \| GU726919 \| GU726920 \| GU726921 \| GU726922 \| GU726923 \| GU726924 \| GU726925 \| \| GU726926 \| GU726927 \| GU726928 \| GU726929 \| GU726930 \| GU726931 \| GU726932 \| GU726933 \| HM053436 \| \| HM053438 \| HM053439 \| HM053440 \| HM053441 \| HM053442 \| HM053443 \| HM053444 \| HM053445 \| HM053446 \| \| HM053447 \| HM053448 \| HM053449 \| HM053450 \| HM053451 \| HM053452 \| HM053453 \| HM053454 \| HM053455 \| \| HM053456 \| HM053457 \| HM053458 \| HM053459 \| HM053460 \| HM053461 \| HM053462 \| HM053463 \| HM053464 \| \| HM053465 \| HM053466 \| HM053467 \| HM130706 \| HQ589218 \| HQ589225 \| JF753554 \| JN099386 \| JN613281 \| \| JN692272 \| JQ520187 \| KF549493 \| KF648564 \| KF963254 \| KF998091 \| KF998092 \| KF998093 \| KF998094 \| \| KF998095 \| KM503134 \| KM655758 \| KP886807 \| KT997431 \| KU194309 \| AY456341 \| FJ463928 \| HM053437 \| \| JN588574 \| JX270802 \| JX270803 \|  \|  \| |
|  | *G. lingzhi* | \| AB811848 \| JQ781855 \| JQ781856 \| JQ781857 \| JQ781858 \| JQ781859 \| JQ781860 \| JQ781861 \| JQ781862 \| \| --- \| --- \| --- \| --- \| --- \| --- \| --- \| --- \| --- \| \| JQ781863 \| JQ781864 \| JQ781865 \| JQ781866 \| JQ781867 \| JQ781868 \| JQ781869 \| JQ781870 \| JQ781871 \| \| JQ781872 \| JQ781873 \| KC222318 \| KC415761 \| KC511557 \| KJ143907 \| KJ143908 \| KR093032 \| KT247417 \| \| KU219990 \| KU219991 \| KU310901 \| KX055521 \| KX055522 \| KX055523 \| KX055524 \| KX055525 \| KX055527 \| \| KX055528 \| KX055531 \| KX055532 \| KX055534 \| KX055536 \| KX055537 \| KX055541 \| KX055546 \| KX055547 \| \| KX055548 \| KX055555 \| KX055562 \| KX055563 \| LC090753 \| LC149597 \| |
|  |  | \| AB811852 \|  \|  \|  \|  \| \| --- \| --- \| --- \| --- \| --- \| |
|  | *G. tsugae* | \| DQ425001 \| DQ425004 \|  \|  \|  \| \| --- \| --- \| --- \| --- \| --- \| |
|  | *G. luteomarginatum* | \| KP226861 \|  \|  \|  \|  \| \| --- \| --- \| --- \| --- \| --- \| |
|  | *Ganoderma* sp. | \| HQ689695 \| HQ689696 \| HQ689697 \| JQ520207 \| JQ520210 \| JQ520211 \| JQ520214 \| \| --- \| --- \| --- \| --- \| --- \| --- \| --- \| \|  \|  \|  \|  \|  \| |
| ***G.* cf. *adspersum*** | *G. adspersum* | \| AM269767 \| AM269770 \| AM269771 \| AM906054 \| AM906055 \| \| AM906056 \| AM906057 \| \| EF060009 \| EF060010 \| \| \| \| --- \| --- \| --- \| --- \| --- \| --- \| --- \| --- \| --- \| --- \| --- \| --- \| --- \| \| EF060011 \| EU162053 \| FJ655447 \| FJ655449 \| FJ655451 \| FJ655452 \| \| FJ655453 \| \| JN176879 \| JN176883 \| \| \| JN176884 \| JN176886 \| JN176887 \| JN176888 \| JN176889 \| JN176890 \| \| JN176891 \| \| JN176892 \| JN176893 \| \| \| \| \| JN176894 \| JN176898 \| JN176901 \| JN176902 \| JN176903 \| JN176904 \| \| JN176905 \| \| JN176906 \| JN176907 \| \| \| \| \| \| JN176908 \| JN222416 \| JN222417 \| JN222418 \| JN588579 \| JN588580 \| \| JN588581 \| \| JN588582 \| JN588583 \| \| \| \| \| \| \| JN588584 \| JN588585 \| JN588586 \| KF605650 \| KF605651 \| KF605652 \| \| KF975893 \| \| KP941436 \| KP941437 \| \| KP941438 \| KP941439 \| KP941440 \| KP941441 \| KP941442 \| X78742/X78763 \| \| \| |
|  |  | \| GU731555 \| FJ655450 \| FJ655448 \| AJ006685 \|  \| \| --- \| --- \| --- \| --- \| --- \| |
|  | *G. australe* | \| AY884182 \| AY884183 \| AY884184 \| FJ655458 \| FJ655459 \| FJ655460 \| FJ655461 \| FJ655462 \| FJ655463 \| \| --- \| --- \| --- \| --- \| --- \| --- \| --- \| --- \| --- \| \| FJ655464 \| FJ655465 \| FR686555 \|  \|  \| \|  \|  \|  \|  \|  \| |
|  | *G. applanatum* | \| GU731554 \| JQ520161 \| KR673486 \| KR673681 \|  \| \| --- \| --- \| --- \| --- \| --- \| |
|  | *Ganoderma* sp. | \| AF255101 \| AF255104 \| AF455397 \| AF455510 \| EF060008 \| EF060012 \| FN548165 \| \| --- \| --- \| --- \| --- \| --- \| --- \| --- \| \|  \|  \|  \|  \|  \| |
|  | Others | \| AY605704 \| AY605708 \| FJ820612 \| KF800469 \| KP974213 \| \| --- \| --- \| --- \| --- \| --- \| |
| ***G.* cf. *applanatum*** | *G. applanatum* | \| AY787672 \| AY884178 \| AY884179 \| FJ655454 \| FJ655455 \| FJ655456 \| FJ655457 \| FR686556 \| GU256764 \| \| --- \| --- \| --- \| --- \| --- \| --- \| --- \| --- \| --- \| \| JN176900 \| JN588587 \| JQ520162 \| JX501311 \| KC505588 \| KC581319 \| KF495001 \| KF605647 \| KF605648 \| \| KF605649 \| KF975891 \| KF975892 \| KJ140577 \| KJ668542 \| KJ857258 \| KJ857259 \| KJ857265 \| KJ857266 \| \| KP941443 \| KR673611 \| KT318588 \| KT318589 \| KU219987 \| KX055561 \| |
|  |  | \| AB811850 \| AJ608709 \| DQ424996 \| DQ425009 \| EU232190 \| EU232219 \| GU213472 \| GU213473 \| GU731554 \| \| --- \| --- \| --- \| --- \| --- \| --- \| --- \| --- \| --- \| \| HM602034 \| HM622152 \| JN008873 \| JQ520161 \| KF494999 \| KM609399 \| KP794597 \| KR673486 \| KR673681 \| \| KR867655 \| KT210090 \| KU194312 \| AB509681 \| AB509663 \| AB509495 \| |
|  | *G. lipsiense* | \| EF059994 \| EF059995 \| EF059996 \| EF059997 \| EF059998 \| EF059999 \| EF060000 \| EF060001 \| EF060002 \| \| --- \| --- \| --- \| --- \| --- \| --- \| --- \| --- \| --- \| \| EF060003 \| EF060004 \| EF060005 \| EF060006 \|  \| |
|  | *G. adspersum* | \| FJ655448 \| FJ655450 \| GU731555 \|  \|  \| \| --- \| --- \| --- \| --- \| --- \| |
|  | *G. oregonense* | \| JQ520196 \|  \|  \|  \|  \| \| --- \| --- \| --- \| --- \| --- \| |
|  | *Ganoderma* sp. | \| AF255092 \| AF255093 \| AF255094 \| AF255095 \| AF255096 \| AF255097 \| JQ520206 \| \| --- \| --- \| --- \| --- \| --- \| --- \| --- \| |
|  | Others | \| FJ609287 \| AY968082 \| EU554851 \| EU554863 \| FJ626936 \| FJ197949 \| FR750674 \| HQ021834 \| HQ021835 \| \| --- \| --- \| --- \| --- \| --- \| --- \| --- \| --- \| --- \| \| HQ021975 \| HQ022119 \| JX675211 \| JX675216 \| KC785577 \| KT334783 \| KT334785 \| KT334788 \| KT334790 \| \| DQ421242 \| GU083147 \| JQ666446 \| JQ666457 \| JQ666465 \| JQ666495 \| JQ666507 \| JQ666577 \| JQ666595 \| \| DQ421100 \|  \|  \|  \|  \| |
| ***G.* cf. *gibbosum*** | *G. gibbosum* | \| AB733121 \| AY593854 \| AY593855 \| AY593856 \| AY593857 \| EU273513 \| EU273514 \| EU273555 \| EU273557 \| \| --- \| --- \| --- \| --- \| --- \| --- \| --- \| --- \| --- \| \| EU326218 \| EU326219 \| EU918695 \| FJ582638 \| KJ195663 \| KR673513 \| KU055650 \| X78741/X78762 \| \| \| |
|  |  | \| FJ392286 \| JN596331 \| JN655531 \| KJ654372 \| KJ654373 \| KJ654404 \| KJ654405 \| KJ654446 \| KT318591 \| \| --- \| --- \| --- \| --- \| --- \| --- \| --- \| --- \| --- \| \| KU194307 \| KU194311 \| KU194326 \| KU194347 \| AY593858 \| |
|  | *G. applanatum* | \| DQ424996 \| DQ425009 \| GU213472 \| GU213473 \| JN008873 \| KF494999 \| KM609399 \| \| --- \| --- \| --- \| --- \| --- \| --- \| --- \| |
|  | *G. australe* | \| GU213474 \| JX195196 \| JX195197 \| JX195199 \| JX195200 \| \| --- \| --- \| --- \| --- \| --- \| |
|  | *G. fulvellum* | \| FJ478088 \|  \|  \|  \|  \| \| --- \| --- \| --- \| --- \| --- \| |
|  | *G. lucidum* | \| AF506372 \|  \|  \|  \|  \| \| --- \| --- \| --- \| --- \| --- \| |
|  | *Ganoderma* sp. | \| AF255105 \| AF255114 \| HQ891299 \|  \|  \| \| --- \| --- \| --- \| --- \| --- \| |

Gray box indicates incorrect sequences.
